# The need for screening, early diagnosis, and prediction of chronic kidney disease in people with diabetes in low- and middle-income countries—a review of the current literature

**DOI:** 10.1186/s12916-022-02438-6

**Published:** 2022-08-02

**Authors:** Cindy George, Justin B. Echouffo-Tcheugui, Bernard G. Jaar, Ikechi G. Okpechi, Andre P. Kengne

**Affiliations:** 1 Non-Communicable Diseases Research Unit, South African Medical Research Council, Francie van Zijl Drive, Parow Valley, PO Box 19070, Cape Town, South Africa; 2 Department of Medicine, Division of Diabetes Endocrinology and Metabolism, Johns Hopkins University School of Medicine, Baltimore, MD USA; 3 Department of Medicine, Division of Nephrology, Johns Hopkins University School of Medicine, Baltimore, MD USA; 4 The Welch Center for Prevention, Epidemiology, and Clinical Research, Baltimore, MD USA; 5 Department of Epidemiology, The Johns Hopkins Bloomberg School of Public Health, Baltimore, MD USA; 6 Nephrology Center of Maryland, Baltimore, MD USA; 7 Division of Nephrology and Hypertension, Groote Schuur Hospital, University of Cape Town, Cape Town, South Africa; 8 Kidney and Hypertension Research Unit, University of Cape Town, Cape Town, South Africa; 9 Division of Nephrology and Immunology, Faculty of Medicine and Dentistry, University of Alberta, Edmonton, AB Canada

**Keywords:** Chronic kidney disease, Diabetes, Screening, Diagnosis, Prediction

## Abstract

Chronic kidney disease (CKD) in people with diabetes is becoming an increasing major public health concern, disproportionately burdening low- and middle-income countries (LMICs). This rising burden is due to various factors, including the lack of disease awareness that results in late referral and the cost of screening and consequent treatment of the comorbid conditions, as well as other factors endemic to LMICs relating to inadequate management of risk factors. We critically assessed the extant literature, by performing searches of Medline via PubMed, EBSCOhost, Scopus, and Web of Science, for studies pertaining to screening, diagnosis, and prediction of CKD amongst adults with diabetes in LMICs, using relevant key terms. The relevant studies were summarized through key themes derived from the Wilson and Jungner criteria. We found that screening for CKD in people with diabetes is generally infrequent in LMICs. Also, LMICs are ill-equipped to appropriately manage diabetes-associated CKD, especially its late stages, in which supportive care and kidney replacement therapy (KRT) might be required. There are acceptable and relatively simple tools that can aid diabetes-associated CKD screening in these countries; however, these tools come with limitations. Thus, effective implementation of diabetes-associated CKD screening in LMICs remains a challenge, and the cost-effectiveness of such an undertaking largely remains to be explored. In conclusion, for many compelling reasons, screening for CKD in people with diabetes should be a high policy priority in LMICs, as the huge cost associated with higher mortality and morbidity in this group and the cost of KRT offers a compelling economic incentive for improving early detection of diabetes in CKD.

## Background

Diabetes mellitus is one of the major public health concerns worldwide, affecting 537 million adults [1]. Although the burden of diabetes is rising globally, it is growing more rapidly in low- and middle-income countries (LMICs) than in high-income countries (HICs); largely driven by population ageing and the rise in obesity prevalence, underpinned by poor nutrition [2] and physical inactivity [3].

A common microvascular complication caused by uncontrolled chronic hyperglycaemia is diabetic nephropathy [4, 5] (also termed diabetic kidney disease [DKD]). DKD occurs in 20–40% of individuals with diabetes [6], generally within 10 years of diabetes onset [7]. Tight glycaemic control, and the control of other risk factors like blood pressure, can prevent the occurrence and progression of microvascular complications in people with diabetes [8, 9]. However, achieving and maintaining optimal risk factor control in LMICs is often challenging.

Given the high cost of care for people with diabetes and chronic kidney disease (CKD), pressure is mounting on LMICs to prioritize screening of these diseases, as kidney replacement therapy (KRT) options are not frequently available or affordable [10]. The huge cost associated with treating advanced CKD offers a compelling economic incentive for improving the early detection of diabetes-associated CKD in LMICs. These would include optimal screening options to detect individuals at-risk of developing CKD, and identifying those with CKD, to reduce the progression to kidney failure or death due to mostly cardiovascular complications [5]. The latter will inevitably require more accurate disease prediction approaches.

This review aims to summarize the available literature on screening for CKD amongst people with diabetes in LMICs. Our review also elaborates on the recent global advances related to screening processes, early diagnosis, and prediction of CKD in people with diabetes, with the aim to place the findings into context and exploring future directions for research.

We searched Medline via PubMed, EBSCOhost, Scopus, and Web of Science for observational studies, randomized controlled trials or reviews pertaining to screening, diagnosis and prediction of CKD amongst adults with diabetes in LMICs, using relevant key terms like “screening”, “diagnosis”, “risk prediction”, “diabetes mellitus”, “type 1 diabetes”, “type 2 diabetes”, “diabetic kidney (renal) disease”, “chronic kidney (or renal) disease”, “kidney (or renal) dysfunction”, “decreased kidney (renal) function”, “end-stage renal (kidney) disease”, “glomerular filtration rate”, “albuminuria”, “proteinuria” “Cockcroft-Gault equation”, “Modification of Diet in Renal Disease equation”, “CKD Epidemiology Collaboration equation”, “prevalence”, “incidence”, and “cardiovascular disease”. Additionally, we hand-searched the reference list of relevant articles and searched websites such as the World Health Organization (WHO) and major global and regional professional organizations in nephrology and cardiovascular diseases (CVDs). The identified entries were evaluated, irrespective of date or country of publication, to retain the most relevant in the judgement of the authors. Since screening processes, early diagnostic tools, and prediction of CKD in populations with diabetes incorporates many different topics which span across various areas of research, the use of a unified selection criteria for inclusion and exclusion of published literature would be challenging. Therefore, rather than being a conventional systematic assessment of every published article addressing the subject, this paper aims to summarize the most relevant literature relating to screening, early diagnosis, and prediction of CKD in individuals with diabetes in the context of LMICs, through a series of key topics derived from the Wilson and Jungner criteria (Table 1) [11].

**Table 1:** Wilson and Jungner criteria for disease screening

1	The condition sought should be an important health problem
2	There should be an accepted treatment for patients with recognized disease
3	Facilities for diagnosis and treatment should be available
4	There should be a latent or early symptomatic stage
5	There should be a suitable test or examination
6	The test should be acceptable to the population
7	The natural history of the condition, including development from latent to declared disease, should be adequately understood
8	There should be an agreed policy on who to treat as patients
9	The cost of case finding (including diagnosis and treatment of patient diagnosis) should be economically balanced in relation to possible expenditure on medical care as a whole
10	Case finding should be a continuing process and not a “once and for all” project

Adapted from the World Health Organization [11]

### Burden of chronic kidney disease in low- to middle-income countries: role of diabetes

The majority (81%) of people with diabetes reside in LMICs [1], with Africa, the Middle East, South East Asia, and Central America expected to experience the highest increase in diabetes cases over the next 10 years [12]. Consequently, the burden of CKD is expected to further increase, in parallel with the rise in diabetes prevalence in these countries.

Nationally representative, population-based data depicting the burden of diabetes-associated CKD is lacking in most LMICs. However, several factors suggest that the prevalence of CKD in people with diabetes is likely greater and more heterogenous in LMICs compared to HICs [13]. According to pooled country data, the prevalence of diabetes-associated CKD is 18% in Ethiopia [14], 23% in Nepal [15], 28% in Nigeria [16], and, based on a large multi-site study including 57,594 people, 53% in Singapore [17]. Studies grouping prevalence rate by continent reported that 22% [18] and 31% of people with diabetes have CKD in Africa (22 countries included) and South Asia (Afghanistan, Bangladesh, Bhutan, Maldives, Nepal, India, Pakistan, and Sri Lanka) [19], respectively. These prevalence rates should however be viewed with caution as most pooled estimates are based on studies with relatively small sample sizes with substantial heterogeneity across the included studies. Although the high and variable prevalence of diabetes-associated CKD in LMICs could be ascribed to factors like the varying population characteristics between countries, and the lack of standardized laboratory procedures and uniform CKD definition, there are various factors specific to LMICs that further exacerbate this problem.

In LMICs, it is common for individuals with CKD to be diagnosed at an advanced stage, due to lack of patient and healthcare provider awareness. A Cameroon-based study found that late-referral to nephrologists occurred in 73.9% of cases, of which 50% were due to the primary care practitioner, 44.8% due to the patients’ lack of awareness, and 5.2% as the combination of both [20]. In Pakistan, although a large proportion of general practitioners recognized diabetes as a risk factor for CKD (88.4%), only 12.5% and 77.2% were aware of the optimal time to screen patients with type 1 and 2 diabetes mellitus (T1DM and T2DM), respectively, for CKD [21]. Due to the lack of awareness, opportunities to prevent adverse outcomes of CKD are severely limited [22] and many people with diabetes progress to kidney failure or succumb due to CVD complications [5, 23, 24].

Availability and excessive out-of-pocket costs associated with treatment are further contributing factors to the lack of risk factor control and prevalent CKD in LMICs. No LMIC has implemented a fully subsidized healthcare program for individuals with non-dialysis CKD [25, 26], which means that in most instances the onus lay solely on the individual to pay out-of-pocket [27, 28]. A prospective epidemiological study of LMICs reported that only 29.6% of participants with diabetes were on treatment, as 27% and 63% of the surveyed households could not afford metformin and insulin, respectively [28]. A recent study found that the cost of diabetes treatment equals 1.2% of the cumulative gross domestic product (US$19.5 billion) in sub-Saharan Africa (SSA), with this cost predicted to rise to between US$35 and US$59 billion by 2030 [29]. The costs associated with blood pressure management further compounds the problem. In the Prospective Urban Rural Epidemiology (PURE) study, 60% of individuals from LMICs found access to β-blockers and angiotensin-converting enzyme inhibitors (ACEi) unaffordable, compared to only 0.1% of households in HICs [30]. The same study also showed that these blood pressure lowering medications were available to only 62% and 37% of urban and rural communities in lower–middle-income countries, and 25% and 3% of urban and rural communities in low-income countries, compared to 95% and 90% in HICs [30]. In another multi-country study including 55 LMICs (37,094 individuals with diabetes), coverage of glucose-lowering medication was 50.5%, antihypertensive medication was 41.3%, and lipid-lowering medication was 6.3%, with HICs having greater coverage [31]. These studies show that fewer than 10% of people with diabetes in LMICs receive coverage of guideline-based comprehensive treatment.

Since there is currently no adequately sized healthcare costing study for people with CKD in LMICs, particularly in the earlier stages of the disease, we made inferences from those conducted in HICs. As highlighted in the review by Jha et al., about 3% of the total healthcare budget in HICs is used to provide treatment for patients with kidney failure, who account for less than 1% of the total population [32]. Treatment costs related to ESKD accounted for 7.2% (US$35.9 billion) of the overall US Medicare paid in 2019 [33], with outpatient and inpatient costs in Taiwan accounting for 10.4–11.1% and 4.8–5.6% of the entire National Health Insurance expenditure, respectively [34]. In 2018, the total medical expenditure related to ESKD was 66 billion New Taiwan dollars [34], with the Netherlands spending between €77,566 and €105,833 of the total healthcare budget on ESKD [35]. The cost of care further increases if care for diabetes is also required. According to the annual US renal report, Medicare spending, which is US$16,112 per patient for those with CKD, is US$19,739 per patient for those with CKD and diabetes [33]. Because only a small fraction of people with CKD reach kidney failure, the economic costs associated with the earlier stages of CKD are even higher and increase as kidney function declines [36]. Indeed, the US spent nearly US$80 billion on CKD (excluding costs associated with ESKD) in 2016 [33], with the estimated cost of CKD to the UK National Health Service in 2010 equalling £1.44 billion [32].

Scaling up the capacity of health systems to deliver treatment not only to lower glucose but also to address other CVD risk factors, such as hypertension and high cholesterol, are key to preventing diabetes-associated CKD in LMICs.

### Chronic kidney disease screening and risk prediction in low- and middle-income countries: reference to diabetes

Currently, the most utilized screening tools for CKD irrespective of aetiology include biochemical testing, and a combination of risk factors in the form of risk prediction models. However, implementing these tools in LMICs come with inherent challenges.

#### Biochemical tests

Identifying and monitoring diabetes-associated CKD primarily involves the assessment of kidney damage (albuminuria) and kidney function (estimated glomerular filtration rate [eGFR]). The general recommendation is that CKD screening in individuals with diabetes should begin within five years after the diagnosis of T1DM and at the diagnosis of T2DM, which should include measuring urinary albumin-to-creatinine ratio (uACR) to detect microalbuminuria and serum creatinine (and/or cystatin C) for the calculation of eGFR [37] (Table 2). These biochemical screening tests have practical advantages and limitations.

**Table 2:** Current screening recommendations for chronic kidney disease in people with diabetes in low- and middle-income countries

When?	Type 1 diabetes mellitus	5 years after diagnosis and annually thereafter [37]
	Type 2 diabetes mellitus	At time of diagnosis and annually thereafter [37]
How?	Spot uACR	Elevated uACR (> 3 mg/mmol) must be confirmed within 3–6 months
	Serum creatinine/cystatin C (eGFR using CKD-EPI equation [38])	Reduced eGFR (< 60 ml/min/1.73m^2^) must be confirmed within 3 months

*Abbreviations*: *uACR* urinary albumin-to-creatinine ratio, *eGFR* estimated glomerular filtration rate, *CKD-EPI* Chronic Kidney Disease Epidemiology Collaboration equation

##### Albuminuria

Albuminuria is a major prognostic factor for CKD progression, morbidity, and mortality from CVD, as it is often the first clinical indicator of CKD in people with diabetes [39]. However, despite albuminuria being a clinically useful tool for predicting prognosis and monitoring response to therapy, there are limitations to its usefulness.

The variability of urinary albumin, the method used to diagnose albuminuria, whether it be urinary albumin excretion rate (uAER), uACR, or by dipstick, has implications in lower-income settings. The assessment of uAER, whether timed or 24-h collections, is a more accurate measure of albumin concentration than uACR; however, it is cumbersome, and the precision of urine collection is questionable, whereas measurement of albumin from spot urine samples, without simultaneously measuring urine creatinine, is less expensive but less accurate. The dipstick test for urinary protein, which is the most frequently used test in LMICs due to its cost-effectiveness and accessibility, is even less accurate, as it is insensitive to reliably detect albumin concentrations in ranges < 300 mg/day, and it correlates poorly with uACR [40].

##### Glomerular filtration rate estimation

The most used estimators of GFR in LMICs are the creatinine-based CKD Epidemiology Collaboration (CKD-EPI) [38] and Modification of Diet in Renal Disease (MDRD) [41] equations; both of which include a race-correction factor. Globally, there has been debate centred on the inclusion of this race-correction factor, due to the potential exacerbation of race-related healthcare disparities [42, 43]. Consequently, the National Kidney Foundation and American Society of Nephrology (NKF-ASN) Task Force recommended to use a raceless equation [42].

Although not widely used in LMICs yet, the inclusion of serum cystatin C in the estimation equations has gained momentum in many HICs, as studies have shown an improved CKD diagnosis and GFR staging [44–46]. However, it remains to be explored across populations, and validation is warranted. For example, a study including 494 participants from the Democratic Republic of Congo and Ivory Coast found no substantial augmented value for cystatin C when used alone or in combination with serum creatinine in CKD-EPI equations [47]. Also, a Pakistan-based study, which included 557 participants, found that eGFR by cystatin C underestimated measured GFR and eGFR by creatinine and cystatin C did not offer substantial advantage compared with eGFR calculated by creatinine [48]. On the contrary, other studies in LMICs have shown that eGFR by cystatin C, either alone or in combination with creatinine, showed better accuracy than equations using creatinine or cystatin C alone [49–51]. It should be noted that most comparative studies of measured versus estimated GFR conducted in LMICs include relatively small samples with even smaller proportions of people with CKD; therefore, the results should be viewed with caution and larger validation studies are required. Also, in most studies from LMICs, GFR is measured with iohexol plasma clearance. Although iohexol plasma clearance is a recognized and accepted reference method, both the CKD-EPI and MDRD have been developed with iothalamate urinary clearance data, which is more cumbersome and costly than plasma clearances. With that being said, the use of creatinine might be favoured above the use of cystatin C at this point, as serum creatinine determination, in addition to it having a well-defined reference standard, is also less expensive (US$0.20/creatinine test using Jaffe reaction vs. US$4/cystatin C test [52]). Also, serial measurements of serum creatinine may be sufficient, provided that they are frequent, as per guidelines [53].

Despite all the concerns related to albuminuria and eGFR and the lack of access to these measures in LMICs [26], there is a need to include these markers in routine practice, as this would give an indication of kidney dysfunction and could lead to earlier referral and intervention [54].

#### Risk prediction models and scores

Several prediction models have been developed to estimate the risk of undiagnosed CKD and risk of progression of CKD in diabetes [55–60]. These risk prediction models have great potential as screening tools, particularly in the context of large-scale population-based screening in LMICs. In practice, CKD diagnosis require at least two biochemical tests on separate occasions at least three months apart [61]. Thus, given the financial implications of continued testing, the ideal scenario would be that a non-invasive risk prediction score would narrow down the proportion of those requiring CKD diagnosis confirmation by eGFR and uACR. Furthermore, the expectation is that the risk score be able to predict the progression of CKD in people with diabetes, which would result in intensifying treatment for those most likely to benefit. To this end, good risk prediction models would be of great advantage in resource-poor settings, as simple and inexpensive prediction models based on demographic, anthropometric, and clinical information already collected during the process of diabetes diagnosis (like HbA1c and blood glucose levels) could reduce the screening burden. However, such risk prediction models have yet to be established and validated in LMICs. The current diabetes-associated CKD prediction models include conventional risk factors like age, gender, ethnicity, in addition to eGFR, uACR, haemoglobin, blood pressure, serum albumin, creatinine, calcium, phosphate, and bicarbonate [58–60, 62], as well as non-conventional factors like certain single-nucleotide polymorphisms [57], troponin T and N-terminal pro-brain natriuretic peptide [63], making them less than ideal for LMICs.

Also, risk models generally tend to be population-specific [64]. According to a systematic review of risk prediction tools, the discriminative performance of prediction models is generally acceptable-to-good in the population in which it was developed and modest-to-acceptable when tested in populations other than the one it was developed in [64]. Currently, only a few of the existing diabetes-specific CKD risk algorithms have been validated in different populations and none in populations of LMICs.

### Potential benefits and challenges in screening for chronic kidney disease in high-risk groups in low- and middle-income countries

The objective of screening is the timely detection of asymptomatic diseases when interventions have a reasonable potential to have a positive impact on outcome. Currently, death in people with diabetes-associated CKD is nearly twice as high as death due to CKD or diabetes alone [65]. Early diagnosis of CKD in people with diabetes will likely prevent CKD development, progression to ESKD and death, in addition to improving quality of life and substantially reducing healthcare cost. Table 3 summarizes the benefits and limitations of screening, early diagnosis, and treatment of CKD in people with diabetes in LMICs.

**Table 3:** Benefits and limitations/barriers of screening, early diagnosis, and treatment of chronic kidney disease in people with diabetes in low- and middle-income countries

Benefits	Limitations/barriers
Detect CKD in the early, asymptomatic stages	•The cost of screening is out of reach for many LMICs •Infrastructure and staff needed for testing is not available in most LMICs •Many LMICs do not have access to laboratory testing in primary care facilities for HbA1c, serum and urinary creatinine and urinary albumin •Many LMICs struggle to treat current (known) CKD cases
Early referral to nephrologist	•Scarcity of kidney care workforce (e.g. nephrologists, renal nurses, dieticians, and social workers) in LMICs •Poorly structured health care delivery systems providing fragmented and interrupted care
Early initiation of treatment	•Excessive out-of-pocket costs are associated with treatment •Access to treatment, including KRT, is limited •Supportive care for people with advanced CKD is non-existent •No LMIC has implemented a fully subsidized healthcare program for individuals with non-dialysis CKD •Newer classes of antidiabetic agents like sodium-glucose cotransporter 2 inhibitors and glucagon-like peptide 1 receptor agonists are unaffordable •Considerable anxiety to patients and families, when effective treatment is not available or is causing economic hardship
Opportunity for intervention to improve prognosis	•Management of detected cases over years or decades is difficult or impossible in most LMICs, due to excessive cost, lack of infrastructure, specialists, etc •Few LMICs would be able to integrate CKD cases identified by screening into the broader health system, as it is already over-burdened

*Abbreviations*: *CKD* chronic kidney disease, *KRT* kidney replacement therapy, *LMICs* low- and middle-income countries

#### Effectiveness of risk factor intervention in low- and middle-income countries—effect on outcomes

Achieving and maintaining tight glycaemic control is essential in managing diabetes to prevent CKD [66]. However, glycaemic control in LMICs has been persistently inadequate over the past decades [3, 67]. In a study of nationally representative surveys from 28 LMICs (47,413 participants), of those with diabetes, only 23% achieved glycaemic control (HbA1c < 6.5%) [68]. In another large observational study from 49 LMICs including 15,079 and 66,088 individuals with T1DM and T2DM, respectively, glycaemic control (HbA1c < 7%) was low (21–38%) and did not improve over a 12-year period (2005–2017) [67]. Hypertension is also a significant consequence and contributor to the development of CKD in diabetes [69]. Although prevalent in T1DM and T2DM, the aetiology differs. Unlike in individuals with T1DM, in which elevated blood pressure is generally due to CKD, in T2DM, hypertension commonly exists prior to the development of albuminuria and reduced eGFR, because of shared risk factors [70]. According to the International Society of Hypertension, around 40% of people over the age of 25 years have hypertension worldwide and two thirds of this population reside in LMICs [71]. Of those with hypertension in LMICs, more than 90% of cases are uncontrolled [72]. Dyslipidaemia, estimated at 34% in LMICs [73, 74], may play a role in the progression of CKD in people with diabetes, but this remains a debated topic [75]. Since the coexistence of CKD and diabetes predicts a greater CVD risk than either condition alone, appropriate management of modifiable cardiovascular risk factors, including dyslipidaemia, is paramount. Consequently, the KDIGO guidelines recommend evaluating the lipid profile of adults with CKD and recommend statin treatment if these individuals also present with diabetes. This recommendation is however not aimed at slowing the progression of CKD per se, but rather to reduce cardiovascular risk [76]. There is also strong evidence for the link between obesity (which is increasing more rapidly in LMICs than in HICs [77]) and CKD in diabetes [78, 79]. It is thought that obesity drives the metabolic derangements, including insulin resistance, hypertension, and dyslipidaemia, all important established risk factors for CKD development and progression [80].

Given the dual burden posed by the progression from CKD to kidney failure and the increased cardiovascular morbidity and mortality, it is imperative to reduce the prevalence of the major risk factors for CKD in people with diabetes. There is ample evidence that early therapeutic intervention for people with diabetes, particularly those aggressively targeting elevated glucose levels and blood pressure, can delay the onset and complications of diabetes, including death due to CVD or ESKD. Indeed, large trials like the United Kingdom Prospective Diabetes Study (UKPDS) [81], Steno-2 [82], Action in Diabetes and Vascular Disease (ADVANCE) [83], and Diabetes Control and Complications Trial (DCCT)/Epidemiology of Diabetes Interventions and Complications (EDIC) trial [84] demonstrated that tight glucose and blood pressure control significantly reduces incidence and progression of CKD in people with T1DM [85] and T2DM [81–83]. Similarly, studies have shown that by tightly regulating blood pressure and lipid levels, early during diabetes development, the rate of decline of eGFR can be attenuated [86–89]. These studies showed that ACEi or angiotensin II receptor blockers (ARBs) reduced the rate of progression to microalbuminuria [86], reduced the progression from microalbuminuria to macroalbuminuria [87, 90], and slowed the development of ESKD in people with diabetes [88, 89].

Recent randomized controlled trials have shown added beneficial effects (beyond only glucose control) of newer classes of antidiabetic agents. Sodium-glucose cotransporter 2 inhibitors (SGLT2i) and glucagon-like peptide 1 (GLP1) receptor agonists have shown to have both cardiovascular and renoprotective effects in patients with T2DM. In the Empagliflozin, Cardiovascular Outcomes, and Mortality in Type 2 Diabetes (EMPA-REG OUTCOME) trial [91], the SGLT2i was associated with an acute, dose-dependent reduction in eGFR of 5 ml/min/1.73 m^2^ and 30–40% reduction in albuminuria. Similarly, in the CANagliflozin cardiovascular Assessment Study (CANVAS), canagliflozin lowered albuminuria with greater proportional reductions in those with moderate-to-severe increased albuminuria, and after 13 weeks, canagliflozin slowed the annual loss of kidney function across albuminuria subgroups, with greater absolute reductions in participants with severely increased albuminuria [92]. Also, results from the Dapagliflozin in Patients with Chronic Kidney Disease (DAPA-CKD) trial, which included CKD patients with and without diabetes, showed that amongst those with CKD, irrespective of diabetes status, the risk of sustained decline in the eGFR of at least 50%, and ESKD or death from kidney or cardiovascular causes was significantly lower in the dapagliflozin-treated group compared to the placebo [93]. In the Liraglutide Effect and Action in Diabetes: Evaluation of Cardiovascular Outcome Results (LEADER) trial [94], the GLP1 analogue liraglutide lowered the risk of new-onset persistent macroalbuminuria, revealing benefits in slowing the development and progression of CKD.

A very small proportion of individuals in LMICs utilizes the novel cardioprotective and renoprotective medications. According to the International Diabetes Management Practices Study (IDMS), which is a large observational study in LMICs, 3.4% of people with T1DM and only 1.0% of those with T2DM are treated with an SGLT2i [95], highlighting the low uptake of these newer agents. This low uptake is mainly driven by the high cost and low accessibility in LMICs. According to a recent cost-effectiveness analysis of second-line therapies for T2DM, the costs of SGLT2i and GLP-1RA would have to be reduced by 17% and 98% for SGLT2i and GLP-1RA, respectively, in order to match the cost-effectiveness of sulfonylureas as second-line treatment [96]. However, LMICs should ensure that there are readily available essential medicines for diabetes mellitus, including generic formulations which are usually more affordable [97].

Furthermore, educational interventions have shown to result in improved patient engagement, use of health services, and adherence. In the Kidney Early Evaluation Program (KEEP) [98], following initial evaluation, 71% of the participants reported visiting a physician during follow-up and those with CKD were 24% more likely to report visiting a physician than those without CKD. This suggests that the program motivated the targeted population to seek further care. Creative strategies are also needed to promote diabetes self-management in lower-income communities. The effectiveness of diabetes self-management education on improving diabetes care and glycaemic control has been demonstrated previously [99, 100]. Indeed, diabetes self-management education programs have been associated with improvement in knowledge, frequency, and accuracy of self-monitoring of blood glucose, self-reported dietary habits, and glycaemic control, particularly in the short term (< 6 months) [99, 100]. Given some of the challenges in LMICs, educational strategies (e.g. community health worker driven lifestyle interventions, methods to improve adherence) could easily be utilized to improve diabetes care and glycaemic control as well as increase kidney disease awareness and improve kidney disease outcomes (e.g. on World Kidney Day).

#### Current screening practices, cost, and cost-effectiveness of screening in low- and middle-income countries

The US National Kidney Foundation recommends the implementation of country-wide screening programs in people with diabetes, in order to detect CKD, at least once yearly using various diagnostic methods [101]. However, in LMICs, people with diabetes are unlikely to be screened for CKD. Findings from the IDMPS showed that people with T2DM were less likely to be screened for CKD (63–89%) compared to those with T1DM (74–91%) [95]. Considering that nearly 80% of all cases of undiagnosed diabetes are from LMICs, one could argue that most, if not all, of these undiagnosed individuals are also unlikely receiving screening for CKD. Thus, when taking into account the total population with diabetes in LMICs (diagnosed and undiagnosed), the screening figures reported in the IDMPS [95] could be reduced quite substantially. Given the increasing prevalence of T2DM and CKD, along with the high rate of undiagnosed diabetes in LMICs, increased investments to detect and treat CKD early should be a policy priority in LMICs [102].

There are many barriers to implementing CKD screening in resource-limited settings related to the cost-effectiveness of such an endeavour. For one, there is little use in screening populations unless follow-up is undertaken, and effective treatment is initiated that improve clinical outcomes, ideally as part of a systematic program. Indeed, screening and detection of CKD cases is probably the easier component, as a large challenge lays in ensuring appropriate management for CKD and diabetes, usually for years or even decades. Screening for CKD in high-risk individuals like people with diabetes, by albuminuria/proteinuria and eGFR, has shown to be cost-effective in HICs [103], with cost-effectiveness ratios ranging between US$5,298 and US$54,943 per quality-adjusted life-year (QALY) for proteinuria screening and US$23,680/QALY by eGFR screening in the population with diabetes [103]. Contrary, there is little data in LMICs showing similar cost-effectiveness. This is largely because access to specialized laboratory facilities is not always readily available at the primary care level in LMICs [25, 26, 104]. In the Global Kidney Health Atlas project [26], only 30% of the 80 LMICs included in the report were able to measure serum creatinine in primary care, and none were able to access eGFR, quantitative urinalysis, uACR, or urine protein-to-creatinine ratio. Further, qualitative urinalysis, using test strips for albumin or protein or both was available in 41% of the LMICs, while 6% had access to HbA1c measurements [26].

Many national health systems in LMICs are not equipped or funded to deliver integrated care for individuals with CKD once screen-detected [25]. Access to treatment, including KRT, is limited in LMICs with significantly fewer individuals receiving dialysis, compared to those in HICs. In a systematic review by Liyanage et al., evaluating global access to treatment for kidney failure, between 20 and 273 individuals/per million in LMICs receive dialysis, compared to 1064 individuals/per million in HICs [105]. This skewed distribution is mainly due to underdiagnosis of CKD in LMICs and affordability of treatment. Governmental financing of KRT is very important, as out-of-pocket payment very often limit the number of patients surviving long enough to receive a KRT. As an example, in a single-centre study in Nigeria, less than 2% of the patients were able to fund dialysis treatments beyond 12 weeks [106]. On the contrary, Thailand is a good example of how increased access resulted in increased rates of dialysis. In 2008, Thailand included treatment for ESKD in their Universal Coverage Scheme, and within 7 years, the incidence and prevalence of dialysis more than tripled [107]. Similarly, the Rwandan government included KRT in the country’s insurance packages and observed an increase in demand for dialysis [108]. Also, in Colombia, the main health care providers (Entidades Promotoras de Salud) are mandated to manage a package of the health plan, which includes dialysis and kidney transplantation, as well as public health activities including screening for diseases such as hypertension, diabetes, and CKD. This type of investment from government suggests that delivery of integrated health care is possible in LMICs [109]. However, with increase access offered as part of Universal Coverage Schemes, governments must prepare for the increase in the use of KRT services. For example, human resources need to be well-developed to avoid heavy workloads and low morale amongst health-care workers caused by an increase in demand after free services are provided [110]. If this process is inadequately managed, this surge in demand might create other barriers to accessing care, such as an increase in waiting times to access care. Further, many national health systems in LMICs are not prioritizing the delivery of integrated care for individuals with CKD post-screening or supportive care for those with advanced CKD, because governments consider other factors like the level of economic development, and the distribution of existing resources toward more prevalent and equally severe diseases in LMICs, such as HIV, tuberculosis, and CVD. Yet, even though preventive measures are important, they cannot mitigate mortality amongst individuals already living with CKD and unable to access appropriate treatment or supportive care, as there are millions of individuals currently in need of treatment for CKD and ESKD who are not receiving it [105].

## Conclusions

A substantial proportion of diabetes-associated CKD can be prevented through primary and secondary interventions geared at disease management. By referring individuals with diabetes to nephrology services early in the disease process, the rate of GFR decline can be reduced. Also, prevention of up-stream risk factors (including socio-economic factors, unhealthy diets, food insecurity (a known risk factor for developing diabetes and poor blood glucose control [111]), tobacco use, and sedentary lifestyle) should be targeted to prevent diabetes-associated CKD in LMICs. Large-scale investments are warranted across the health system to support diabetes management and consequent CKD prevention and management. Given the high prevalence of CKD in people with diabetes and the exorbitant cost of treatment of both diseases, the accessibility to treatment in LMICs remains low compared with HICs. There is an obvious need to better characterize the burden of CKD in people with diabetes in LMIC settings. Which leads to the question “which strategies to adopt in LMICs?” Diagnosis and treatment options are generally available in hospitals and health care centres; however, people are not always aware of the disease and do not seek treatment early in the disease progression. Also, even though old and new agents have shown significant benefits in kidney outcomes, cardiovascular outcomes, and mortality, cost and availability of drugs is a never-ending issue in many LMICs [112]. Although there is no simple answer to this question, we should invest in solutions to address this challenge.

## Data Availability

Data sharing is not applicable to this article as no datasets were generated or analysed during the current study. This is a review and all data referred to in this manuscript is publicly available.

## Abbreviations

ACE: Angiotensin-converting enzyme
ADVANCE: Action in Diabetes and Vascular Disease
ARB: Angiotensin II receptor blocker
CANVAS: CANagliflozin cardiovascular Assessment Study
CKD: Chronic kidney disease
CKD-EPI: CKD Epidemiology Collaboration
CVD: Cardiovascular disease
DAPA-CKD: Dapagliflozin in Patients with Chronic Kidney Disease
DCCT/EDIC: Diabetes Control and Complications Trial/Epidemiology of Diabetes Interventions and Complications
DKD: Diabetic kidney disease
eGFR: Estimated glomerular filtration rate
EMPA-REG OUTCOME: Empagliflozin, Cardiovascular Outcomes, and Mortality in Type 2 Diabetes
GLP1: Glucagon-like peptide 1
HICs: High-income countries
IDMPS: International Diabetes Management Practices Study
KRT: Kidney replacement therapy
LEADER: Liraglutide Effect and Action in Diabetes: Evaluation of Cardiovascular Outcome Results
LMICs: Low- and middle-income countries
MDRD: Modification of Diet in Renal Disease
NKF-ASN: National Kidney Foundation and American Society of Nephrology
PURE: Prospective Urban Rural Epidemiology
QALY: Quality-adjusted life-year
SAMRC: South African Medical Research Council
SGLT2i: Sodium-glucose cotransporter 2 inhibitors
T1DM: Type 1 diabetes mellitus
T2DM: Type 2 diabetes mellitus
uACR: Urinary albumin-to-creatinine ratio
uAER: Urinary albumin excretion rate
UKPDS: United Kingdom Prospective Diabetes Study
